# Efficacy of Selenium Supplementation in Graves’ Orbitopathy: A Systematic Review and Meta-Analysis of Randomized Controlled Trials with Trial Sequential Analysis

**DOI:** 10.3390/jcm15124710

**Published:** 2026-06-17

**Authors:** Nikolay Kostadinov, Zlatko Kirovakov, Plamen Penchev

**Affiliations:** 1 Faculty for Public Health and Health Care, Burgas State University “Prof. Dr. Asen Zlatarov”, 8010 Burgas, Bulgaria; kirovakov@yahoo.com; 2 Faculty of Medicine, Medical University of Plovdiv, 4002 Plovdiv, Bulgaria

**Keywords:** selenium, supplementation, Graves’ orbitopathy, thyroid eye disease, meta-analysis

## Abstract

**Background:** Selenium (Sel) supplementation has been proposed as an antioxidant adjunct in Graves’ orbitopathy (GO), with early randomized evidence suggesting benefits in quality of life (QoL), ocular involvement, and disease progression in mild GO. However, subsequent trials across populations with different Sel status and disease severity have yielded inconsistent findings. This systematic review and meta-analysis of randomized controlled trials (RCTs) reassessed the efficacy of Sel supplementation in GO. **Methods:** PubMed, Scopus, and the Cochrane Library were searched from inception to 1 May 2026 for RCTs, comparing Sel supplementation with placebo or no Sel supplementation in patients with GO (PROSPERO “CRD420261395074”). Heterogeneity was assessed using I^2^ statistics and Cochran’s Q test. Risk ratios (RRs) were calculated using the Mantel–Haenszel method, and mean differences (MDs) using the Inverse-Variance method. Random-effects models with restricted maximum-likelihood estimation were applied. **Results:** Five RCTs including 303 patients were analyzed, of whom 165 (56%) received Sel. Sel supplementation was associated with a significant reduction in clinical activity score (MD −1.05; 95% CI −1.61 to −0.48; I^2^ = 52%; *p* < 0.01). No significant differences were observed in palpebral aperture (MD −0.12; 95% CI −1.22 to 0.98; I^2^ = 58%; *p* = 0.83), although this anatomical parameter should be interpreted cautiously because it may be influenced by thyroid functional status and hyperthyroidism-related Müller muscle hyperfunction. No significant differences were observed in QoL improvement (RR 1.72; 95% CI 0.43 to 6.92; I^2^ = 86%; *p* = 0.24) or visual function (MD 6.31; 95% CI −1.40 to 14.03; I^2^ = 45%; *p* = 0.11). **Conclusions:** Sel supplementation may improve clinical activity score in patients with Graves’ orbitopathy, but this finding should be interpreted cautiously given the small number of trials, limited sample size, and clinically relevant heterogeneity. Current evidence does not show consistent benefits for palpebral aperture, quality of life, or visual function. Larger RCTs stratified by baseline Sel status and disease severity are needed before firm conclusions can be drawn.

## 1. Introduction

Graves’ orbitopathy (GO) is an immune-mediated inflammatory disorder of the orbit and periorbital tissues and represents the most common extrathyroidal manifestation of Graves’ disease. Even mild GO can substantially impair vision-related functioning, appearance-related well-being, and overall quality of life (QoL), while a subset of patients may progress to more active or severe diseases, requiring immunosuppressive or rehabilitative treatment. Oxidative stress has been implicated in the pathogenesis of Graves’ disease and GO, providing a biological rationale for antioxidant-based interventions such as selenium (Sel) supplementation [1,2].

Sel is an essential trace element incorporated into selenoproteins with antioxidant and immunomodulatory functions [2,3]. The pivotal randomized controlled trial (RCT) by Marcocci et al. reported that 6 months of Sel improved GO-specific QoL, reduced ocular involvement, and slowed progression in patients with mild GO, supporting its incorporation into European guidance for mild, active GO, particularly in Sel-deficient areas [1]. Subsequent studies expanded the evidence base but introduced uncertainty: a Mexican randomized study suggested improved clinical activity and reduced progression in mild GO, whereas trials in Sel-sufficient settings and in inactive moderate-to-severe GO have reported more heterogeneous effects [4,5,6].

Despite increasing clinical use, several questions remain unresolved. Current recommendations are largely influenced by limited trial evidence, and prior syntheses have not fully clarified whether benefits differ by baseline Sel status, GO activity/severity, treatment duration, or outcome domain. Moreover, conventional meta-analysis may yield statistically significant pooled effects before the accumulated sample size is sufficient, increasing the risk of random error. Therefore, an updated synthesis incorporating trial sequential analysis (TSA) is needed to determine whether the available randomized evidence is conclusive or whether further adequately powered trials are required. This systematic review and updated meta-analysis aimed to evaluate the efficacy and safety of Sel supplementation in patients with GO and to assess the robustness of the cumulative evidence using TSA.

## 2. Materials and Methods

### 2.1. Protocol Registration and Reporting Standards

This systematic review and meta-analysis followed the Cochrane Handbook for Systematic Reviews of Interventions and the Preferred Reporting Items for Systematic Reviews and Meta-Analyses (PRISMA) 2020 statement; the completed PRISMA checklist is provided as Table S1 in the Supplementary Materials [7,8]. This meta-analysis did not require Institutional Review Board approval because it used data from previously published and publicly available articles. This systematic review and meta-analysis was registered with the International Prospective Register of Systematic Reviews (PROSPERO) under the ID “CRD420261395074”.

### 2.2. Eligibility Criteria

Studies that met all the following criteria were included in the meta-analysis: (1) adult patients diagnosed with GO; (2) Sel-containing supplementation or therapy; (3) placebo, standard care, or control without Sel supplementation; (4) any reported clinical, functional, radiological, or patient-reported outcomes; (5) RCTs; (6) no restrictions regarding duration of follow-up or publication period. Studies were excluded if they met one of the following criteria: (1) pediatric populations (<18 years) or included patients without GO; (2) did not evaluate a Sel-containing intervention; (3) lacked an appropriate comparator group; (4) did not provide sufficient data for extraction or analysis; (5) overlapping populations; (6) case reports or series, editorials, letters, conference abstracts without full-text availability, as well as animal or in vitro models.

### 2.3. Search Strategy and Data Extraction

We systematically searched PubMed, Scopus, and Cochrane Central from inception to 1 May 2026 with the following search strategy: (“Graves Ophthalmopathy” [Mesh] OR “Graves orbitopathy” OR “Graves ophthalmopathy” OR “thyroid eye disease” OR “thyroid-associated ophthalmopathy” OR “thyroid associated ophthalmopathy” OR “thyroid orbitopathy” OR “endocrine orbitopathy” OR “dysthyroid orbitopathy”) AND (“Selenium” [Mesh] OR selenium OR “selenium supplementation” OR “selenium supplement” OR “selenium supplements” OR “sodium selenite” OR selenomethionine) AND (“Randomized Controlled Trial” [Publication Type] OR “Controlled Clinical Trial” [Publication Type] OR “randomized controlled trial” OR “randomised controlled trial” OR “randomized” OR “randomised” OR “trial”). Restrictions were applied to only English-language articles and gray literature was excluded. We manually searched the references of all included studies to identify any additional studies. Two authors (N.K. and P.P.) independently extracted the data using predefined search criteria, quality assessment methods, and Rayyan software version 4.3.1 [9]. Any disagreements between these authors were resolved through consensus.

### 2.4. Outcomes and Subgroup Analyses

This meta-analysis included clinical activity scores, QoL, visual functioning, and palpebral aperture as endpoints. Additionally, we conducted a subgroup analysis based on disease severity.

### 2.5. Quality Assessment

The risk of bias was assessed using the Cochrane Collaboration’s tool for assessing the risk of bias in randomized studies of interventions (RoB2) [10]. The RoB2 tool categorizes the risk of bias as low, some concerns, or high. Two authors (N.K. and Z.K.) independently performed the assessments, resolving disagreements through consensus. Publication bias was evaluated using contour-enhanced funnel plots with the trim-and-fill method, which allows for a better interpretation of asymmetry related to statistical significance thresholds. Additional methods, such as p-curve or p-uniform analysis, were not feasible due to the absence of reported exact *p*-values or test statistics in all included studies. Following the Cochrane guidelines, the Egger test was not performed because fewer than 10 studies were included in the meta-analysis [8].

### 2.6. Statistical Analysis

Risk ratios (RRs) with 95% confidence intervals (CIs) were computed for binary outcomes to compare effects for binary endpoints using the Mantel–Haenszel method [11]. Mean differences (MDs) with 95% CI were pooled for continuous outcomes with the Inverse-Variance method [12]. The restricted maximum-likelihood estimator random-effects model was applied for all outcomes to account for heterogeneity and small sample sizes [13,14]. Heterogeneity was assessed using the I^2^ statistic and the Cochran Q test. Two-sided *p*-values < 0.05 were regarded as statistically significant. Subgroup analyses were performed based on disease severity to minimize the risk of selection bias. Leave-one-out (LOO) sensitivity analyses were also conducted to assess the robustness of the findings. A Baujat plot was generated to identify studies contributing most to heterogeneity and their influence on the overall meta-analysis results. This diagnostic tool visually represents the balance between a study’s contribution to heterogeneity (*x*-axis) and its weight in the me-ta-analysis (*y*-axis), aiding in the interpretation of outlier or highly influential studies. Statistical analysis was performed using R software version 4.3.1 with the packages “metafor” and “meta” [15].

### 2.7. Trial Sequential Analysis

The required information size (RIS) was calculated using a two-sided type I error of 5% and power of 80%. For continuous outcomes, the anticipated intervention effect was based on the pooled effect estimate observed in the included trials. Heterogeneity correction was applied using a random-effects model to account for between-study variability [16].

## 3. Results

### 3.1. Study Selection and Baseline Characteristics

**Figure 1:**
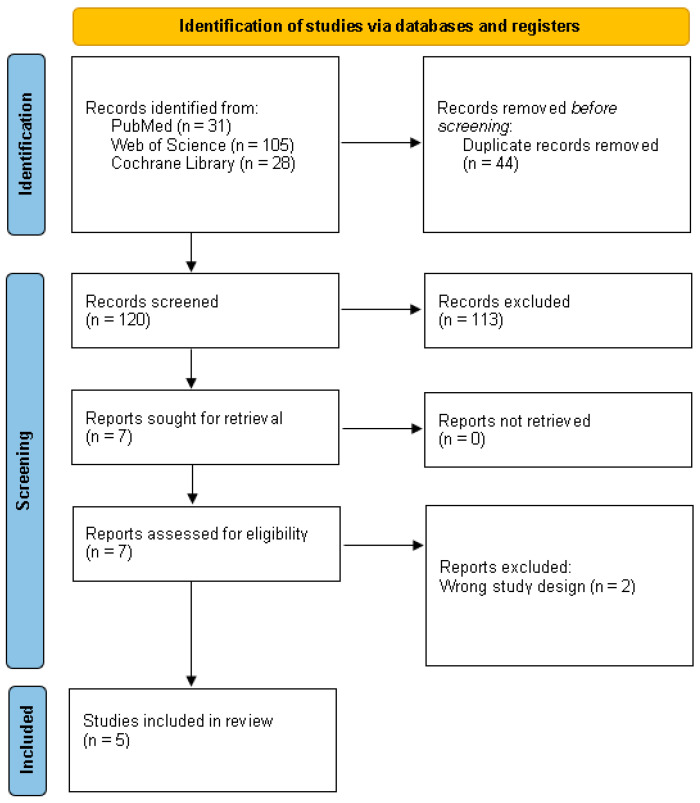
PRISMA flow chart and study selection.

The search strategy yielded a total of 164 results. After removing duplicate records and unrelated articles or abstracts, the remaining 7 studies were fully reviewed to determine whether they met the inclusion and exclusion criteria (Figure 1). Five studies were included, with 303 patients, of whom 165 (54%) received Sel [1,4,5,6,17]. The mean age of the patients in the Sel group was 42.2 years, and of the control group 44.2 years. Study characteristics are presented in Table 1. Wang et al. (2024) [17] was included because, despite being titled as a prospective controlled cohort study, the full-text Methods reported random allocation to Sel supplementation or control.

### 3.2. Pooled Analyses of the Included Studies

#### 3.2.1. Clinical Activity Score

**Figure 2:**
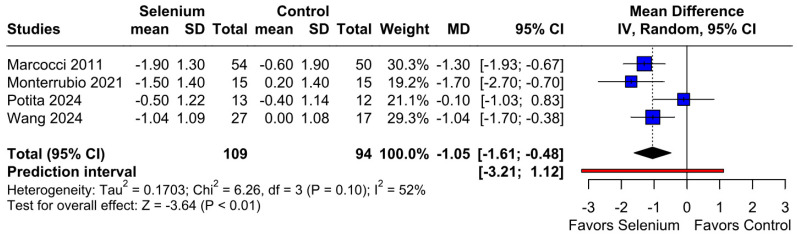
Forest plot of the effect of Sel on clinical activity score. Sel was associated with a significant reduction in clinical activity score compared with control, although moderate heterogeneity and a wide prediction interval suggest that the result should be interpreted cautiously [1,4,5,17].

**Figure 3:**
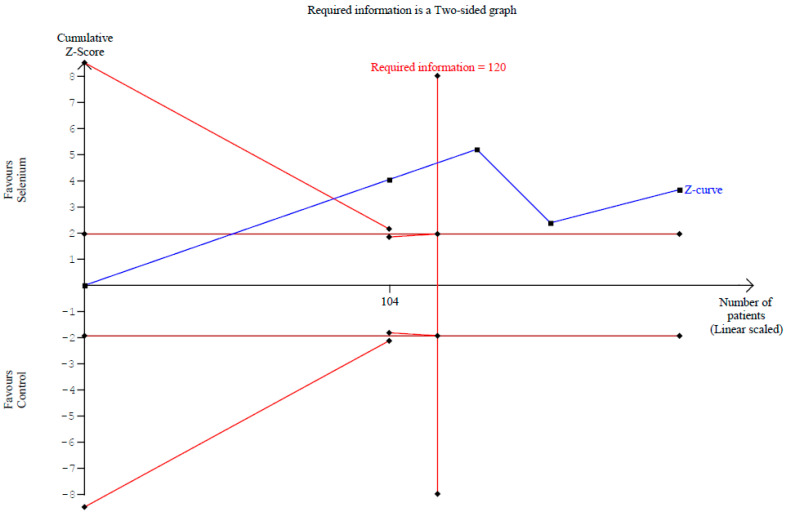
TSA for clinical activity score. TSA showed a significant difference favoring Sel, as the cumulative Z-curve crossed the significance boundary and reached the required information size. However, this finding should be interpreted cautiously due to the limited number of included studies.

Sel supplementation was associated with a greater reduction in clinical activity score compared with control (MD −1.05; 95% CI −1.61 to −0.48; *p* < 0.01), favoring Sel. However, this result should be interpreted cautiously because heterogeneity was moderate (I^2^ = 52%) (Figure 2). TSA showed a significant difference favoring Sel, as the cumulative Z-curve crossed the significance boundary and reached the required information size. However, this finding should be interpreted cautiously due to the limited number of included studies (Figure 3). LOO sensitivity analysis showed that the pooled effect remained significant and consistently favored Sel after omitting each study individually. The effect estimates ranged from MD −0.89 to −1.27, suggesting that no single study disproportionately influenced the overall result (Figure S1). The Baujat plot indicated that Potita 2024 [5] contributed the most to overall heterogeneity and had the greatest influence on the pooled result (Figure S2).

#### 3.2.2. Palpebral Aperture

**Figure 4:**
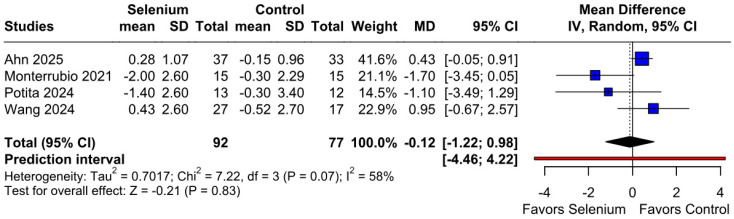
Forest plot of the effect of Sel on palpebral aperture. Sel was not associated with a significant improvement in palpebral aperture compared with control, with moderate heterogeneity [4,5,6,17].

Sel was not associated with a significant change in palpebral aperture compared with control (MD −0.12; 95% CI −1.22 to 0.98; *p* = 0.83). Heterogeneity was moderate (I^2^ = 58%), suggesting substantial uncertainty and variable effects across studies (Figure 4). LOO sensitivity analysis showed that the pooled effect remained non-significant after omitting each study individually. The direction and magnitude of the effect varied across exclusions, suggesting some instability and that no consistent benefit of Sel on palpebral aperture was demonstrated (Figure S3). The Baujat plot suggested that Monterrubio 2021 [4] contributed most to overall heterogeneity, while Ahn 2025 [6] had the greatest influence on the pooled result (Figure S4).

#### 3.2.3. QoL

**Figure 5:**
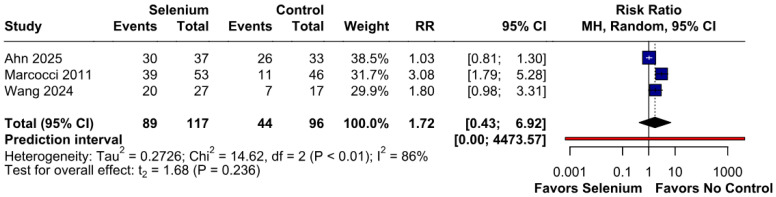
Forest plot of the effect of Sel on QoL. Sel was not associated with a statistically significant improvement in QoL compared with control, with substantial heterogeneity and a very wide prediction interval indicating considerable uncertainty across studies [1,6,17].

Sel was not associated with a statistically significant improvement in QoL compared with control (RR 1.72; 95% CI 0.43 to 6.92; *p* = 0.236). Heterogeneity was substantial (I^2^ = 86%), and the prediction interval was extremely wide, indicating considerable uncertainty and variability across studies (Figure 5). LOO sensitivity analysis showed that the pooled QoL effect remained non-significant after omitting each study individually. The wide confidence intervals and high heterogeneity indicate that the result is unstable and should be interpreted cautiously (Figure S5). The Baujat plot indicated that Potita 2024 [5] contributed most to overall heterogeneity and had the greatest influence on the pooled QoL result (Figure S6).

#### 3.2.4. Visual Function

**Figure 6:**
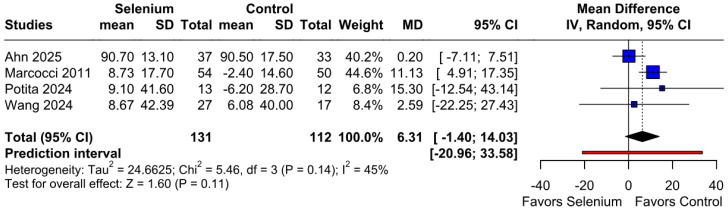
Forest plot of the effect of Sel on visual function. Sel was not associated with a statistically significant improvement in visual function compared with control, with moderate heterogeneity and a wide prediction interval indicating considerable uncertainty and variability across studies [1,5,6,17].

Sel was not associated with a statistically significant improvement in visual function compared with control (MD 6.31; 95% CI −1.40 to 14.03; *p* = 0.11). Heterogeneity was moderate (I^2^ = 45%), and the wide prediction interval (−20.96 to 33.58) suggests considerable uncertainty and variability across studies (Figure 6). LOO sensitivity analysis showed that the pooled effect for visual function was generally non-significant after omitting individual studies. However, omission of Ahn 2025 [6] resulted in a significant effect, suggesting some instability and that the overall result may be influenced by this study (Figure S7). The Baujat plot indicated that Ahn 2025 [6] contributed most to overall heterogeneity, while Marcocci 2011 [1] had the greatest influence on the pooled visual function result (Figure S8).

### 3.3. Subgroup Analyses

#### 3.3.1. Disease Severity

##### Clinical Activity Score

In the subgroup analysis by GO severity, Sel significantly reduced clinical activity score in the mild GO subgroup (MD −1.41; 95% CI −1.95 to −0.88; I^2^ = 0%). The mild-to-moderate subgroup also favored Sel based on a single study (MD −1.04; 95% CI −1.70 to −0.38), whereas the moderate-to-severe subgroup showed no significant benefit (MD −0.10; 95% CI −1.03 to 0.83). The test for subgroup differences was borderline significant (*p* = 0.05), suggesting that treatment effects may vary according to disease severity (Figure S9).

##### Palpebral Aperture

In the subgroup analysis by GO severity, Sel was not associated with a significant overall improvement in palpebral aperture (MD −0.12; 95% CI −1.22 to 0.98). The mild-to-moderate subgroup showed a significant effect favoring control (MD 0.47; 95% CI 0.02 to 0.93; I^2^ = 0%), while the mild and moderate-to-severe subgroups were based on single studies and showed non-significant effects. Subgroup differences were significant (*p* = 0.03), suggesting that effects may differ according to disease severity (Figure S10).

##### QoL

In the subgroup analysis by GO severity, Sel was not associated with a significant overall improvement in QoL (RR 1.75; 95% CI 0.44 to 6.95). The mild GO subgroup showed a significant benefit favoring Sel (RR 3.08; 95% CI 1.79 to 5.28), while the mild-to-moderate subgroup was non-significant with substantial heterogeneity (I^2^ = 71%). Subgroup differences were significant (*p* = 0.02), suggesting that QoL effects may vary according to GO severity (Figure S11).

##### Visual Functioning

In the subgroup analysis by GO severity, Sel was not associated with a significant overall improvement in visual functioning (MD 6.31; 95% CI −1.40 to 14.03). The mild GO subgroup showed a significant effect (MD 11.13; 95% CI 4.91 to 17.35), whereas the mild-to-moderate subgroup showed no significant benefit (MD 0.39; 95% CI −6.62 to 7.41; I^2^ = 0%). The moderate-to-severe subgroup was based on a single study and was also non-significant. Subgroup differences were not statistically significant (*p* = 0.07), although a possible trend by disease severity was observed (Figure S12).

### 3.4. Risk of Bias and Publication Bias Assessment

**Figure 7:**
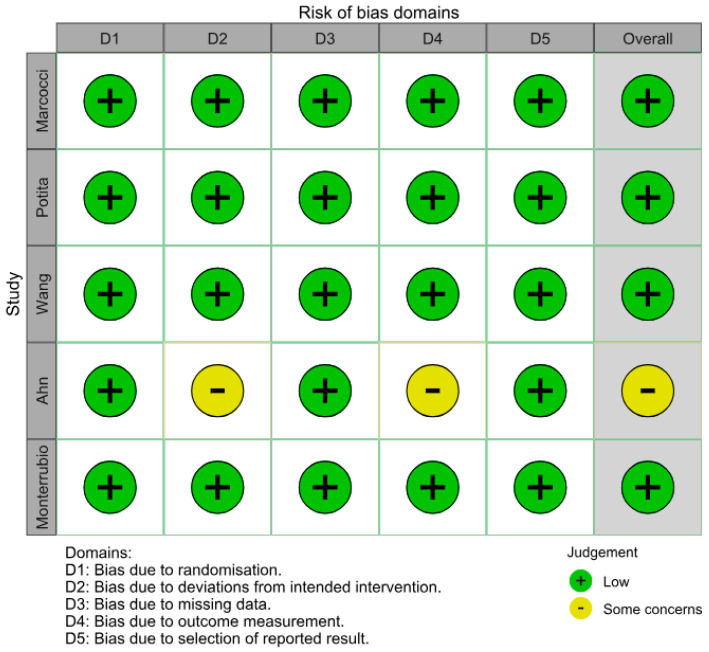
Risk of bias assessment summary [1,4,5,6,17].

Among the five included RCTs, four were assessed as having low risk of bias, and one as having some concerns for bias based on the RoB2 tool. The detailed evaluation is presented in Figure 7. Publication bias was evaluated using contour-enhanced trim-and-fill funnel-plot analyses, plotting individual study weights against point estimates. The funnel plots did show some asymmetry, but given the small number of included studies, visual interpretation is limited (Figures S13–S16).

## 4. Discussion

This systematic review and meta-analysis suggests that Sel supplementation may reduce disease activity in GO, but its effects on anatomical and patient-reported outcomes remain uncertain. The main finding was a significant reduction in CAS favoring Sel, with a pooled MD of −1.05. This supports the biological rationale that Sel, through antioxidant and immunomodulatory selenoproteins, may attenuate orbital inflammation in GO. Oxidative stress has long been implicated in the pathogenesis of Graves’ disease and GO, and Sel may reduce inflammatory activity by improving redox balance and modulating immune responses [1,2].

Our findings are partly consistent with the pivotal EUGOGO trial by Marcocci et al., which showed that 6 months of Sel improved GO-specific quality of life, reduced ocular involvement, and slowed disease progression in patients with mild GO [1]. This trial strongly influenced current European recommendations, which support Sel supplementation in mild active GO, particularly in Sel-deficient areas [2]. In our analysis, the benefit on CAS was most evident in patients with mild GO, whereas evidence was weaker or absent in mild-to-moderate and moderate-to-severe disease. This suggests that Sel may be most effective in early, mild, inflammatory disease rather than in more advanced or inactive orbitopathy, where fibrotic or structural changes may be less responsive to antioxidant therapy.

The timing of Sel initiation may also be an important determinant of treatment response. Sel is generally expected to be more effective when administered early in the inflammatory phase of GO, before chronic fibrotic or structural orbital changes become established. However, disease duration or eye symptom duration was not consistently reported across the included studies, with some trials not providing this information [4,17]. This limits our ability to determine whether differences in timing of Sel supplementation contributed to the observed heterogeneity. Future RCTs should therefore report disease duration in detail and evaluate whether early initiation of Sel leads to greater clinical benefit.

However, the overall certainty of benefit should be interpreted cautiously. Although the pooled CAS result was statistically significant and trial sequential analysis suggested that the cumulative evidence crossed the significance boundary and reached the required information size, only a small number of trials were included. Moreover, moderate heterogeneity was present, and the prediction interval crossed the null, indicating that future studies may observe smaller or even absent effects. The leave-one-out analysis supported the stability of the CAS result, but the Baujat plot identified Potita 2024 as an important contributor to heterogeneity, highlighting that differences in disease severity, activity status, population characteristics, and Sel status may influence treatment effects [4,5,6].

In contrast to CAS, Sel was not associated with significant improvement in palpebral aperture, QoL, or visual function in the overall analyses. This is clinically important because symptomatic improvement in GO is multidimensional and does not always parallel changes in inflammatory activity. Palpebral aperture reflects eyelid position and periocular anatomy, which may be influenced by chronic tissue remodeling rather than active inflammation alone. Similarly, QoL and visual function are complex outcomes affected by appearance, diplopia, ocular discomfort, visual acuity, psychosocial distress, and baseline disease burden. The non-significant pooled results for QoL and visual function, despite positive findings in earlier mild-GO studies, suggest that Sel’s benefits may not be uniform across outcome domains or disease stages [1,4,6].

The interpretation of palpebral aperture requires particular caution. Although palpebral aperture is a clinically relevant anatomical outcome in GO, it may be influenced not only by orbital inflammation but also by thyroid functional status. In hyperthyroid patients, sympathetic overactivity and Müller muscle hyperfunction may contribute to eyelid retraction and increased palpebral aperture, independently of Sel intake. Therefore, variability in thyroid status across trials may have confounded the observed effects on palpebral aperture. Because thyroid functional status was not consistently reported across the included studies, the effect of Sel on this outcome should be considered uncertain.

The interpretation of QoL and visual function outcomes is further limited by differences in measurement instruments across studies. QoL in GO may be assessed using disease-specific questionnaires, such as the GO-QoL, or broader patient-reported outcome measures, while visual function may reflect different domains, including visual acuity, diplopia, ocular discomfort, or vision-related functioning. Because these instruments capture different aspects of patient experience and orbital disease, pooling them may introduce clinical and methodological heterogeneity. This likely contributed to the substantial heterogeneity observed for QoL and the wide uncertainty around visual function estimates.

Subgroup findings further support this interpretation. For QoL and visual function, benefits appeared more pronounced in mild GO, whereas results were not significant in broader or more severe subgroups. This aligns with the hypothesis that Sel is more likely to be useful as an adjunctive therapy in early inflammatory disease, rather than as a treatment for established moderate-to-severe or inactive GO [17].

Sel effect may also depend on baseline Sel status. Khong et al. found that serum Sel concentrations were lower in patients with Graves’ orbitopathy than in patients with Graves’ disease without orbitopathy, supporting the hypothesis that relative Sel insufficiency may contribute to GO susceptibility or severity [18]. Similarly, Lumyongsatien et al. reported that relative Sel insufficiency was associated with more severe GO, further supporting Sel status as a potential effect modifier rather than a universally applicable treatment target [19]. This is relevant to the discrepancy between the positive European and Mexican trials and the more neutral findings from Sel-sufficient settings [1,4,6].

Thyroid status may also influence both the clinical course of GO and the biological response to Sel supplementation. Sel is involved in thyroid hormone metabolism through selenoproteins, including iodothyronine deiodinases, and thyroid dysfunction may alter oxidative stress, immune activity, and Sel requirements. Therefore, whether patients were euthyroid, hyperthyroid, hypothyroid, or had undergone thyroidectomy could represent an important effect modifier. However, thyroid functional status and previous thyroidectomy were not consistently reported across the included trials, preventing subgroup analysis according to these variables. This should be considered an important limitation, and future RCTs should systematically report thyroid status, thyroid-directed treatments, and previous thyroidectomy when evaluating Sel supplementation in GO.

The broader endocrine literature also supports a cautious interpretation. Lanzolla et al. reviewed Sel in Graves’ disease and GO and emphasized that the evidence is strongest for mild GO, while data for more severe disease or Sel-sufficient populations remain limited [20]. Recent analyses also suggest that Sel may have time-dependent or subgroup-specific effects rather than consistent benefits across all GO outcomes. For example, Sung et al. reported that baseline Sel status was associated with treatment response in thyroid eye disease, indicating that future trials should stratify participants according to Sel levels [21]. Finally, the GRASS trial in Graves’ hyperthyroidism showed that Sel supplementation did not clearly improve remission rate or QoL when added to antithyroid drugs, suggesting that Sel effects may be disease-domain specific and should not be extrapolated broadly across Graves’ disease manifestations [22].

The recent randomized trial by Ahn et al. in a Sel-sufficient region found limited benefits of Sel compared with control, raising the possibility that baseline Sel status may be a key effect modifier [6]. This may partly explain discrepancies between European studies, where Sel deficiency is more common, and trials conducted in Sel-sufficient populations.

The substantial heterogeneity observed for QoL and the wide confidence and prediction intervals across several outcomes indicate that the current evidence remains fragile. Differences in trial design, GO severity, baseline Sel status, outcome definitions, follow-up duration, and measurement instruments likely contributed to between-study variability. In addition, some subgroup analyses were based on single studies, limiting the reliability of severity-specific conclusions. Therefore, subgroup findings should be viewed as hypothesis-generating rather than definitive.

The heterogeneity observed across the included studies represents a major limitation of the present evidence base and substantially affects the certainty and generalizability of the pooled findings. Although all included studies were randomized trials, they differed in important clinical and methodological aspects, including GO severity and activity, baseline Sel status of the study populations, Sel dose, outcome definitions, and follow-up duration. These differences may explain why Sel appeared to reduce inflammatory disease activity, particularly CAS, while effects on palpebral aperture, QoL, and visual function were inconsistent. Therefore, the pooled estimates should not be interpreted as evidence of a uniform treatment effect across all patients with GO. Rather, the findings suggest that Sel may be more beneficial in selected patients, particularly those with mild active disease and possible Sel insufficiency. Because the limited number of trials precluded more robust subgroup analyses or meta-regression, these observations should be considered hypothesis-generating.

This study has several strengths, including restriction to randomized evidence, use of random-effects modeling, LOO sensitivity analyses, Baujat diagnostics, and TSA to assess the robustness of cumulative evidence. Nevertheless, important limitations remain. First, the number of included trials was small, limiting statistical power and precluding reliable assessment of publication bias. Second, some outcomes were reported inconsistently across trials, and several analyses included few participants. Third, Sel status was not uniformly available, preventing formal subgroup analysis by deficiency or sufficiency. Fourth, thyroid functional status, previous thyroidectomy, and thyroid-directed treatment were not consistently reported across studies, although these factors may influence Sel metabolism and GO activity. Finally, clinical heterogeneity was substantial, particularly regarding GO activity and severity.

Overall, Sel supplementation appears to provide a modest but statistically significant reduction in CAS, particularly in mild GO. However, current randomized evidence does not demonstrate consistent benefit for palpebral aperture, QoL, or visual function. These findings support a cautious, individualized use of Sel, especially in patients with mild active GO and possible Sel deficiency, while emphasizing the need for larger, adequately powered RCTs stratified by Sel status, GO activity, and disease severity.

## 5. Conclusions

This systematic review and meta-analysis of 5 RCTs and 303 patients suggests that Sel supplementation may reduce clinical activity score in Graves’ orbitopathy, particularly in mild disease. However, the evidence remains limited by the small number of trials, modest sample size, and clinically relevant heterogeneity. Sel did not show consistent benefits for palpebral aperture, QoL, or visual function. Larger RCTs stratified by baseline Sel status, disease activity, and severity are needed to clarify which patients are most likely to benefit.

## Figures and Tables

**Table 1:** Baseline characteristics of the included studies.

Characteristics/Study	Wang et al. 2024 [17] *	Potita et al. 2024 [5]	Marcocci et al. 2011 [1]	Monterrubio et al. 2021 [4]	Ahn et al. 2025 [6]
Study design	RCT	RCT	RCT	RCT	RCT
Country	Multicenter	Thailand	China	Mexico	Korea
No. Patients	74	25	104	30	70
Sel	46	13	54	15	37
Control group (C)	28	12	50	15	33
Age *	Sel: 42.76	Sel: 42.2	Sel: 43	Sel: 40.7	Sel: 40.8
	C: 44.14	C: 48.1	C: 44.6	C: 42.5	C: 42.9
Females n (%)	Sel: 30 (65%)	Sel: 6 (46%)	Sel: 48 (89%)	Sel: 12 (80%)	Sel: 31 (84%)
	C: 17 (61%)	C: 9 (75%)	C: 41 (82%)	C: 11 (73%)	C: 24 (73%)
Smokers n (%)	Sel: 20 (43%)	Sel: 2 (15%)	Sel: 2 (15%)	NR	Sel: 2 (5%)
	C: 20 (71%)	C: 2 (16%)	C: 2 (16%)		C: 5 (15%)
Dosage	Sel: 100 μg twice daily	Sel: 200 μg twice daily	Sel: 100 μg twice daily	Sel: 100 μg twice daily	Sel: 100 μg twice daily
	C: 100 μg twice daily	C: 200 μg twice daily	C: 100 μg twice daily	C: 100 μg twice daily	C: 100 μg twice daily
Duration of treatment	6 months	6 months	6 months	6 months	6 months
Eye symptoms duration	NR	8 months	8 months	NR	8 months
Severity of orbitopathy	Mild-to-moderate	Moderate-to-severe	Mild	Mild	Mild-to-moderate
Follow-up	5 years	6 months	1 year	6 months	5 years

* Although Wang et al. (2024) [17] is titled as a prospective controlled cohort study, the full-text Methods report random allocation to Sel supplementation or control; therefore, it was considered eligible for inclusion as a randomized study.

## Data Availability

No new data were created or analyzed in this study.

## Supplementary Materials

The following supporting information can be downloaded at: https://www.mdpi.com/article/10.3390/jcm15124710/s1, Figure S1. LOO sensitivity analysis for clinical activity score. The pooled effect remained significant and consistently favored selenium after sequential omission of each study, suggesting that no single study disproportionately influenced the overall result; Figure S2. Baujat plot for clinical activity score. Potita 2024 contributed most to overall heterogeneity and had the greatest influence on the pooled effect, while the remaining studies showed lower to moderate influence; Figure S3. LOO sensitivity analysis for palpebral aperture. The pooled effect remained non-significant after sequential omission of each study, indicating no consistent benefit of Sel on palpebral aperture and suggesting some instability in the effect estimates; Figure S4. Baujat plot for palpebral aperture. Monterrubio 2021 contributed most to overall heterogeneity, while Ahn 2025 had the greatest influence on the pooled result; Figure S5. LOO sensitivity analysis for QoL. The pooled QoL effect remained non-significant after sequential omission of each study, with wide confidence intervals and substantial heterogeneity; Figure S6. Baujat plot for QoL. Potita 2024 contributed most to overall heterogeneity and had the greatest influence on the pooled QoL result; Figure S7. LOO sensitivity analysis for visual function. The pooled effect was generally non-significant after sequential omission of individual studies; however, exclusion of Ahn 2025 resulted in a significant effect, suggesting some instability in the overall estimate; Figure S8. Baujat plot for visual function. Ahn 2025 contributed most to overall heterogeneity, while Marcocci 2011 had the greatest influence on the pooled visual function result; Figure S9. Subgroup analysis by GO severity for clinical activity score. Sel showed the clearest benefit in patients with mild GO, while no significant improvement was observed in the moderate-to-severe subgroup; subgroup differences were borderline significant; Figure S10. Subgroup analysis by GO severity for palpebral aperture. Sel was not associated with a significant overall improvement in palpebral aperture, while subgroup differences were significant, indicating possible variation in treatment effects according to GO severity; Figure S11. Subgroup analysis by GO severity for QoL. Sel showed a significant QoL benefit in patients with mild GO, while the overall effect was non-significant and highly heterogeneous, indicating uncertainty and possible variation by disease severity; Figure S12. Subgroup analysis by GO severity for visual functioning. Sel showed a significant improvement in the mild GO subgroup, while the overall effect was non-significant and subgroup differences did not reach statistical significance, indicating uncertainty regarding variation by disease severity; Figure S13. Contour-enhanced trim-and-fill funnel plot for CAS. The plot illustrates individual study weights against point estimates; Figure S14. Contour-enhanced trim-and-fill funnel plot for palpebral aperture. The plot illustrates individual study weights against point estimates; Figure S15. Contour-enhanced trim-and-fill funnel plot for QoL. The plot illustrates individual study weights against point estimates; Figure S16. Contour-enhanced trim-and-fill funnel plot for visual functioning. The plot illustrates individual study weights against point estimates. Table S1. PRISMA 2020 Checklist.
