# Supplementary material for: Efficacy of Selenium Supplementation in Graves’ Orbitopathy: A Systematic Review and Meta-Analysis of Randomized Controlled Trials with Trial Sequential Analysis
Source: J Clin Med. 2026 Jun 17;15(12):4710. doi: 10.3390/jcm15124710 (PMC13302020; doi:10.3390/jcm15124710)
Supplement: Supplementary file 1 [file jcm-15-04710-s001.zip › jcm-4347692-supplementary.pdf]

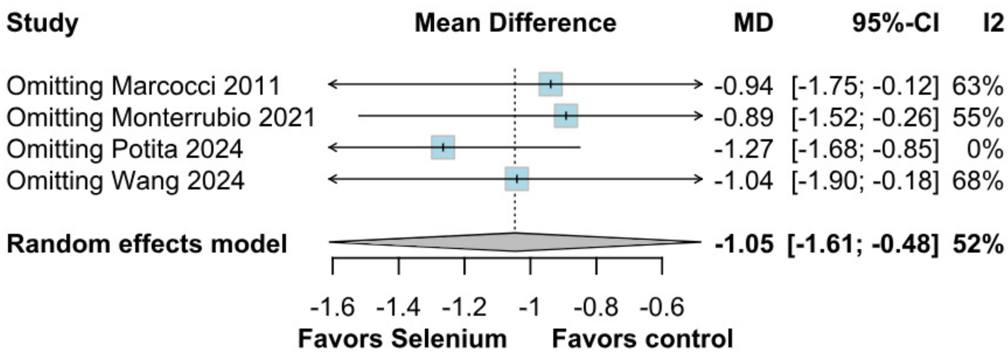

Figure S1. LOO sensitivity analysis for clinical activity score. The pooled effect remained significant and consistently favored selenium after sequential omission of each study, suggesting that no single study disproportionately influenced the overall result.

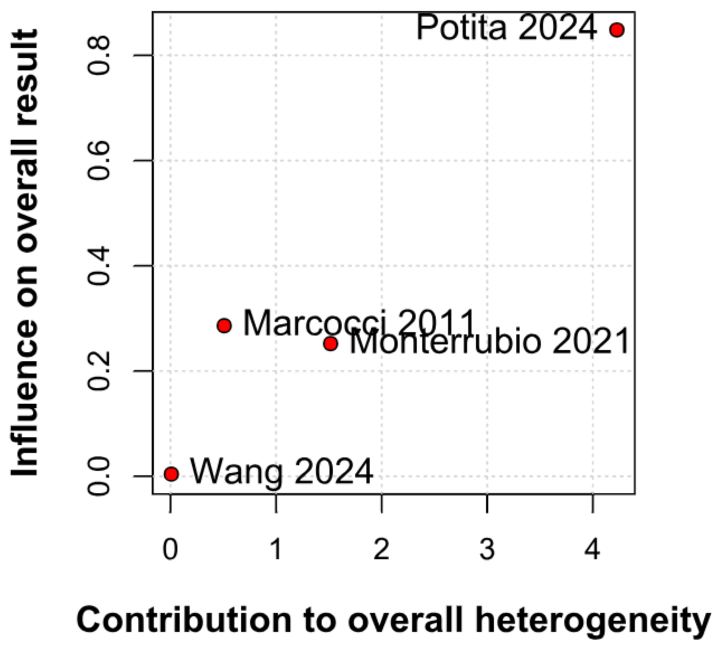

Figure S2. Baujat plot for clinical activity score. Potita 2024 contributed most to overall heterogeneity and had the greatest influence on the pooled effect, while the remaining studies showed lower to moderate influence.

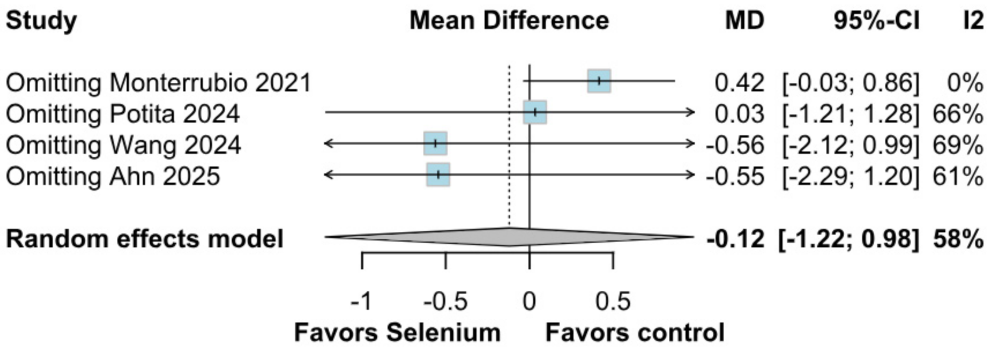

Figure S3. LOO sensitivity analysis for palpebral aperture. The pooled effect remained non-significant after sequential omission of each study, indicating no consistent benefit of Sel on palpebral aperture and suggesting some instability in the effect estimates.

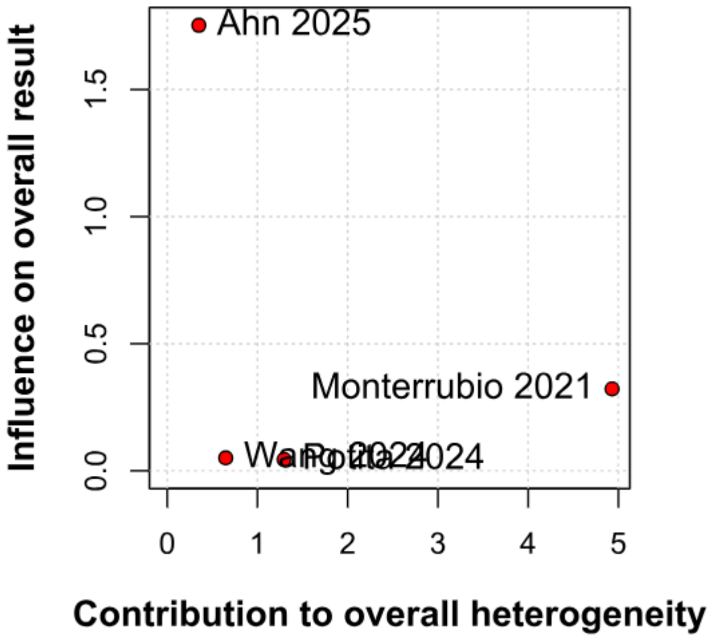

Figure S4. Baujat plot for palpebral aperture. Monterrubio 2021 contributed most to overall heterogeneity, while Ahn 2025 had the greatest influence on the pooled result.

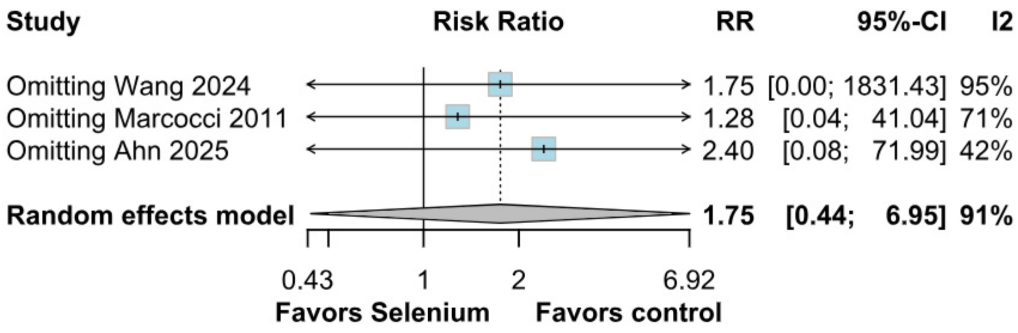

Figure S5. LOO sensitivity analysis for QoL. The pooled QoL effect remained non-significant after sequential omission of each study, with wide confidence intervals and substantial heterogeneity.

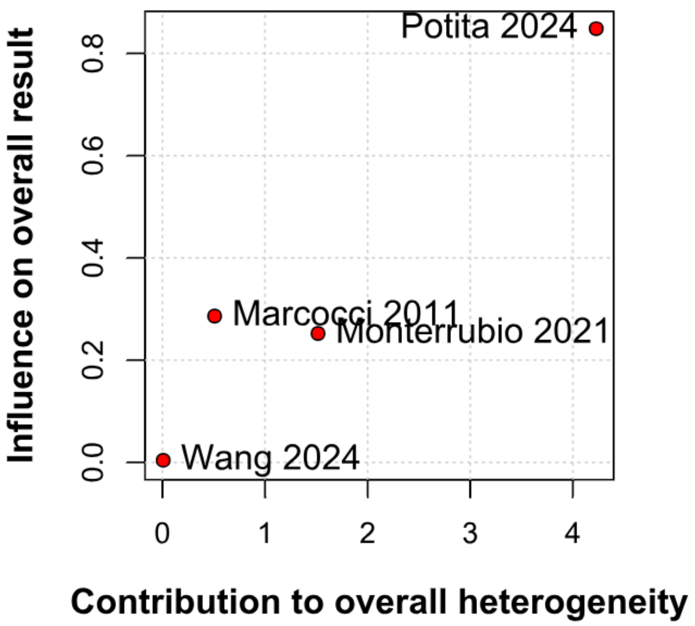

Figure S6. Baujat plot for QoL. Potita 2024 contributed most to overall heterogeneity and had the greatest influence on the pooled QoL result.

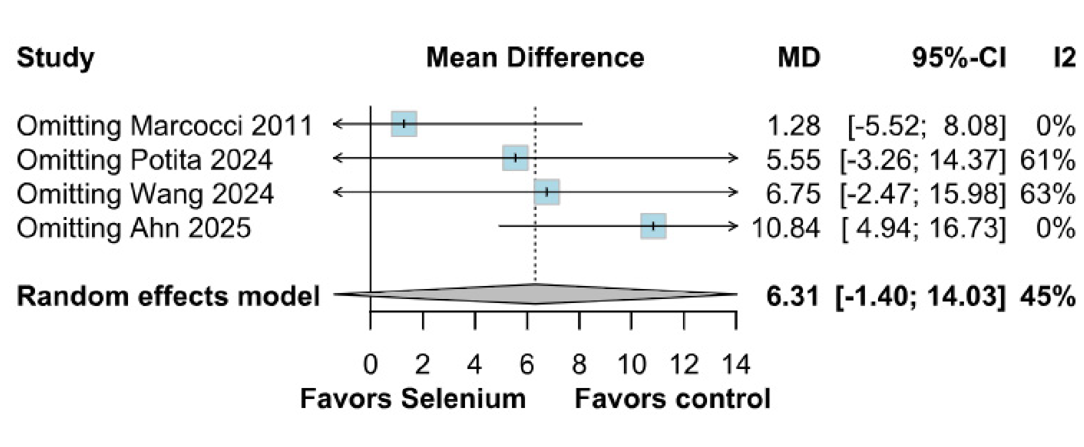

Figure S7. LOO sensitivity analysis for visual function. The pooled effect was generally non-significant after sequential omission of individual studies; however, exclusion of Ahn 2025 resulted in a significant effect, suggesting some instability in the overall estimate**.**

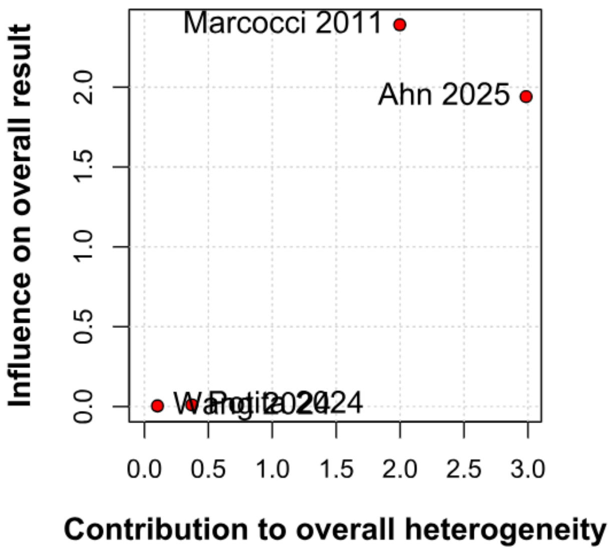

Figure S8. Baujat plot for visual function. Ahn 2025 contributed most to overall heterogeneity, while Marcocci 2011 had the greatest influence on the pooled visual function result

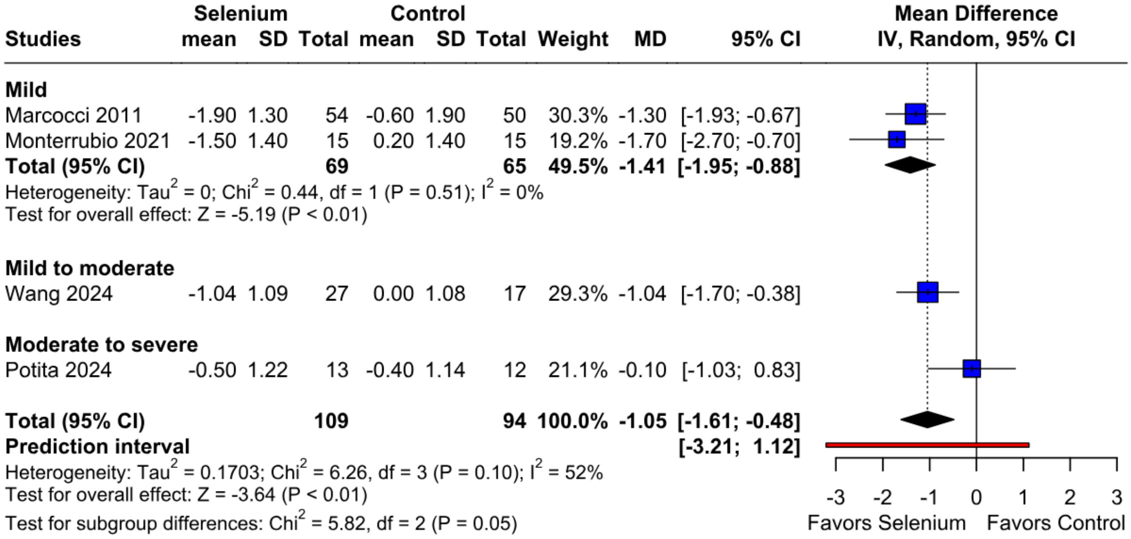

Figure S9. Subgroup analysis by GO severity for clinical activity score. Sel showed the clearest benefit in patients with mild GO, while no significant improvement was observed in the moderate-to-severe subgroup; subgroup differences were borderline significant.

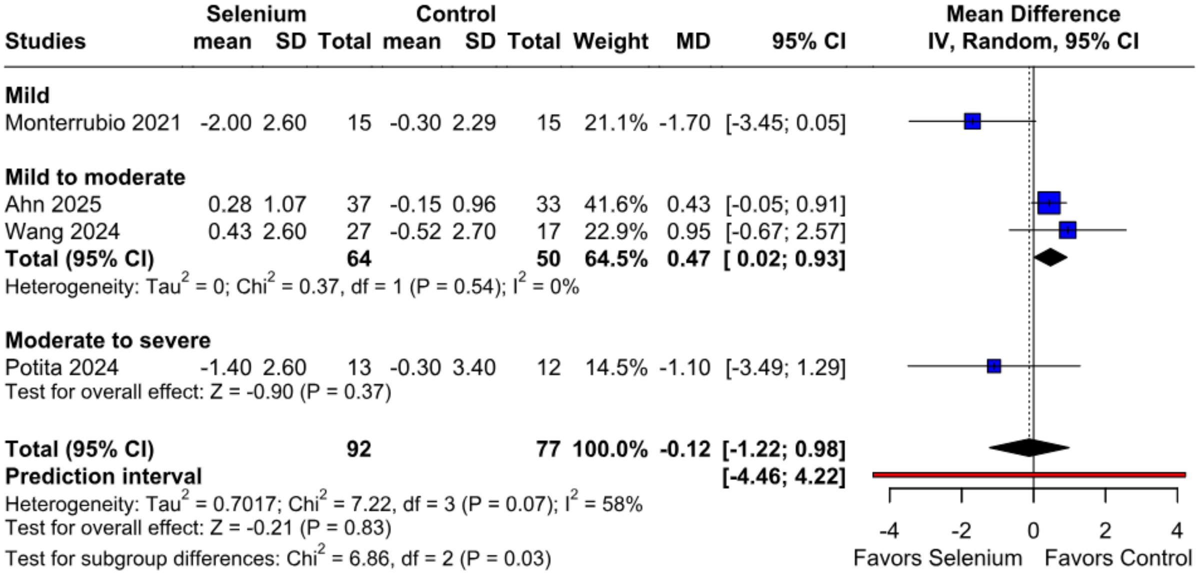

Figure S10. Subgroup analysis by GO severity for palpebral aperture. Sel was not associated with a significant overall improvement in palpebral aperture, while subgroup differences were significant, indicating possible variation in treatment effects according to GO severity.

Study or Subgroup	Selenium Events	Selenium Total	Control Events	Control Total	Weight	RR	95% CI
**Mild**							
Marcocci 2011	39	53	11	46	32.4%	3.08	[1.79; 5.28]
Test for overall effect: t_{Inf} = 4.08 (P < 0.001)							
**Mild to moderate**							
Ahn 2025	30	37	26	33	36.3%	1.03	[0.81; 1.30]
Wang 2024	20	27	7	17	31.3%	1.80	[0.98; 3.31]
**Total (95% CI)**	**50**	**64**	**33**	**50**	**67.6%**	**1.28**	**[0.04; 41.04]**
Heterogeneity: Tau^2 = 0.1373; Chi^2 = 3.46, df = 1 (P = 0.06); I^2 = 71%							
**Total (95% CI)**	**89**	**117**	**44**	**96**	**100.0%**	**1.75**	**[0.44; 6.95]**
**Prediction interval**							**[0.00; 69550.51]**
Heterogeneity: Tau^2 = 0.5060; Chi^2 = 21.22, df = 2 (P < 0.01); I^2 = 91%							
Test for overall effect: t_2 = 1.74 (P = 0.224)							
Test for subgroup differences: Chi^2 = 5.10, df = 1 (P = 0.02)							

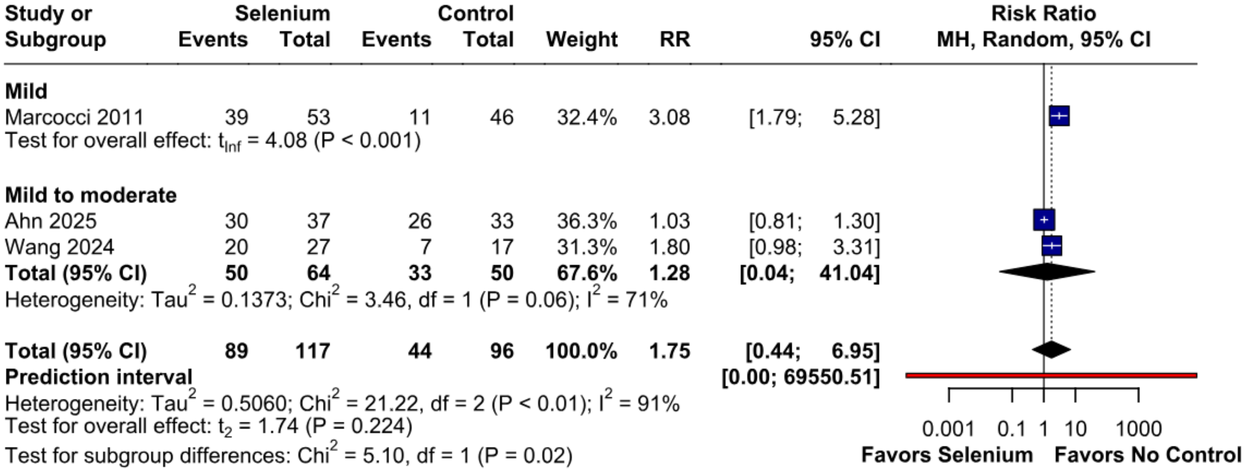

Figure S11. Subgroup analysis by GO severity for QoL. Sel showed a significant QoL benefit in patients with mild GO, while the overall effect was non-significant and highly heterogeneous, indicating uncertainty and possible variation by disease severity.

Studies	Selenium mean	Selenium SD	Selenium Total	Control mean	Control SD	Control Total	Weight	MD	95% CI
**Mild**									
Marcocci 2011	8.73	17.70	54	-2.40	14.60	50	44.6%	11.13	[4.91; 17.35]
**Mild to moderate**									
Ahn 2025	90.70	13.10	37	90.50	17.50	33	40.2%	0.20	[-7.11; 7.51]
Wang 2024	8.67	42.39	27	6.08	40.00	17	8.4%	2.59	[-22.25; 27.43]
**Total (95% CI)**			**64**			**50**	**48.5%**	**0.39**	**[-6.62; 7.41]**
Heterogeneity: Tau^2 = 0; Chi^2 = 0.03, df = 1 (P = 0.86); I^2 = 0%									
**Moderate to severe**									
Potita 2024	9.10	41.60	13	-6.20	28.70	12	6.8%	15.30	[-12.54; 43.14]
Test for overall effect: Z = 1.08 (P = 0.28)									
**Total (95% CI)**			**131**			**112**	**100.0%**	**6.31**	**[-1.40; 14.03]**
**Prediction interval**									**[-20.96; 33.58]**
Heterogeneity: Tau^2 = 24.6625; Chi^2 = 5.46, df = 3 (P = 0.14); I^2 = 45%									
Test for overall effect: Z = 1.60 (P = 0.11)									
Test for subgroup differences: Chi^2 = 5.42, df = 2 (P = 0.07)									

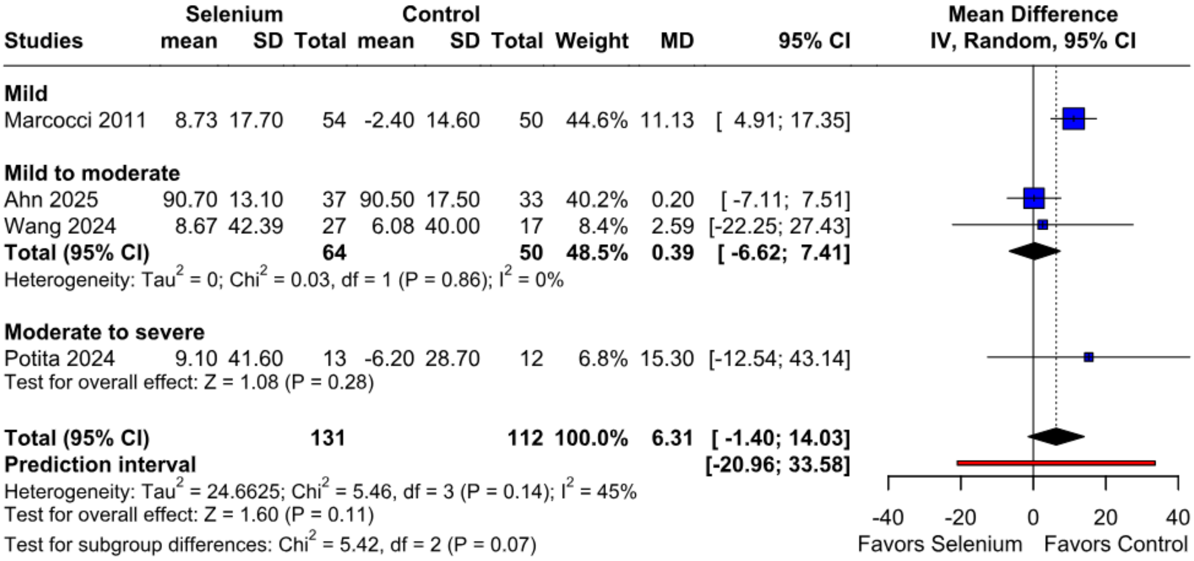

Figure S12. Subgroup analysis by GO severity for visual functioning. Sel showed a significant improvement in the mild GO subgroup, while the overall effect was non-significant and subgroup differences did not reach statistical significance, indicating uncertainty regarding variation by disease severity.

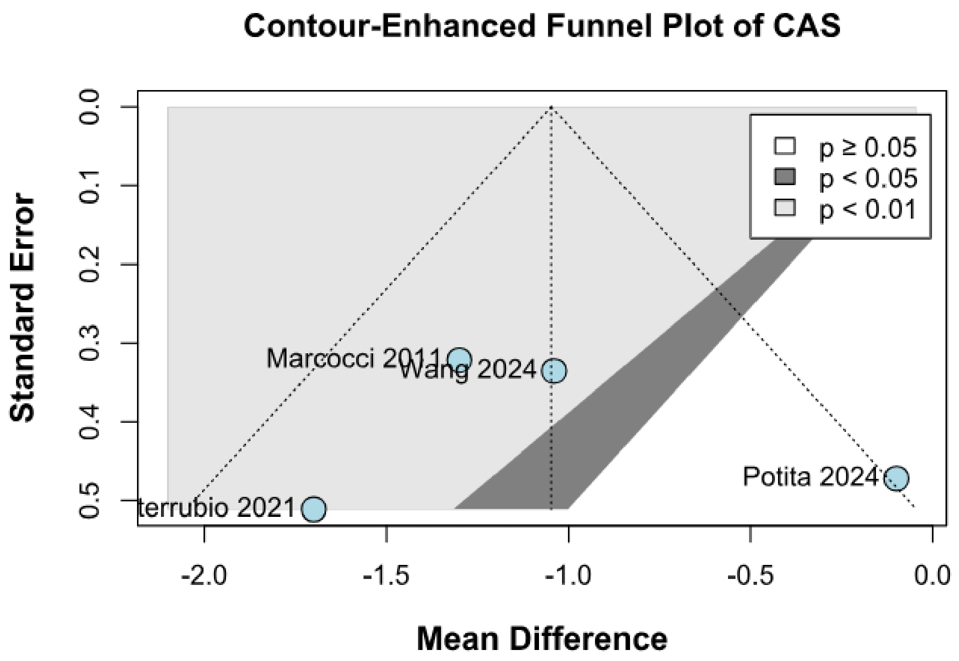

Figure S13. Contour-enhanced trim-and-fill funnel plot for CAS. The plot illustrates individual study weights against point estimates

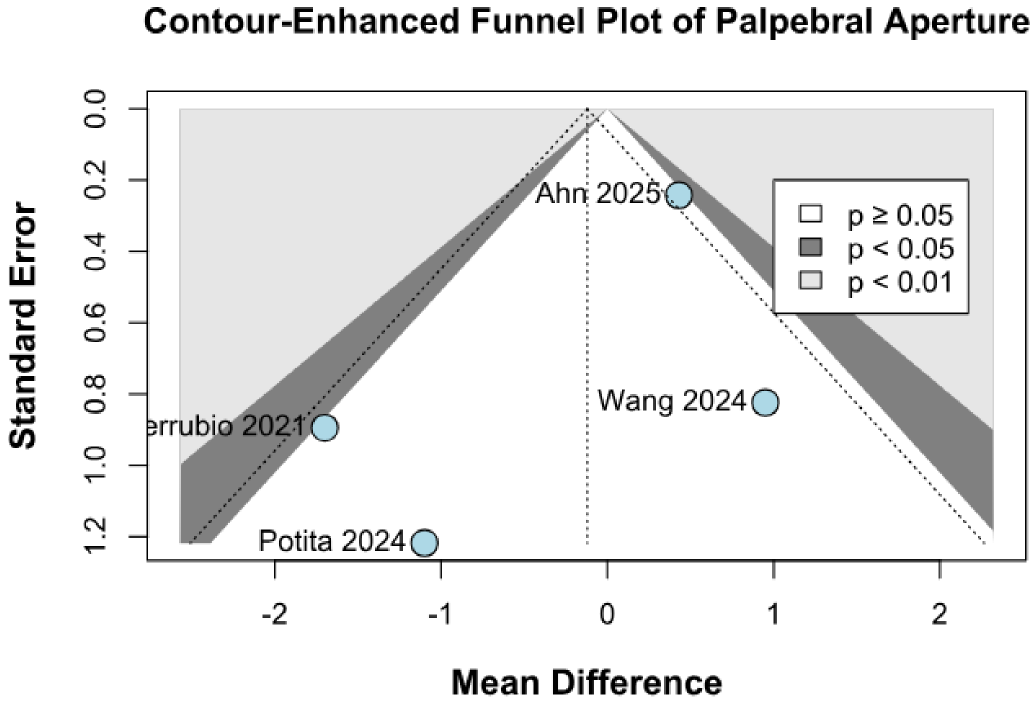

Figure S14. Contour-enhanced trim-and-fill funnel plot for palpebral aperture. The plot illustrates individual study weights against point estimates

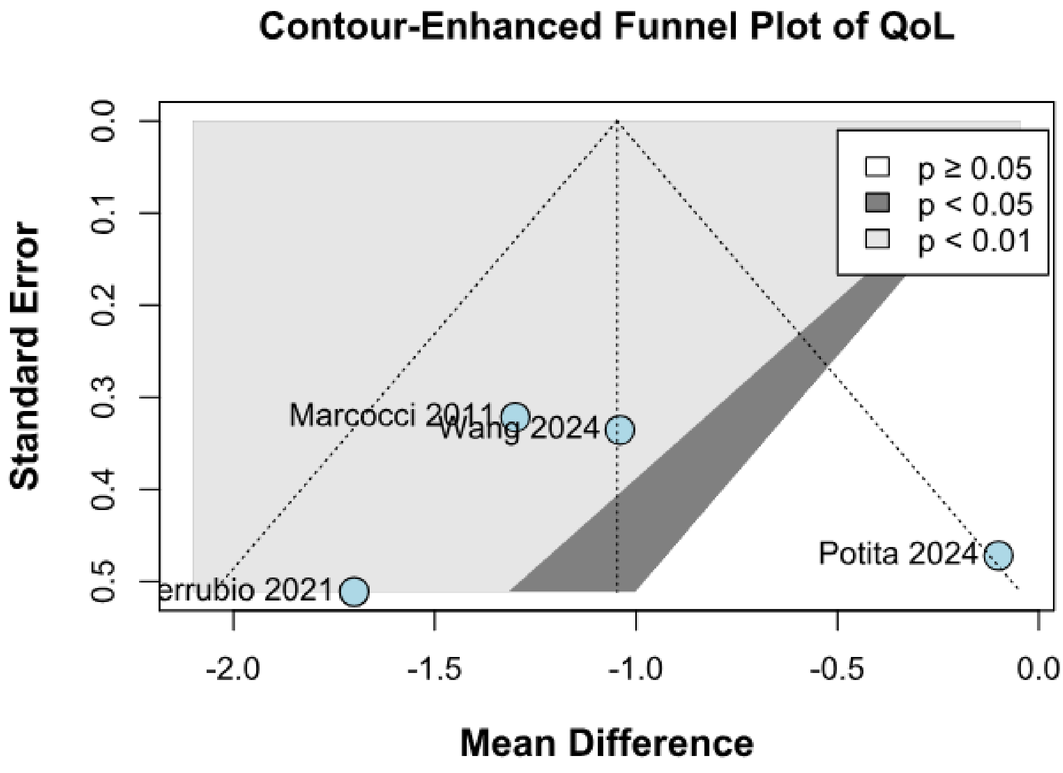

Figure S15. Contour-enhanced trim-and-fill funnel plot for QoL. The plot illustrates individual study weights against point estimates

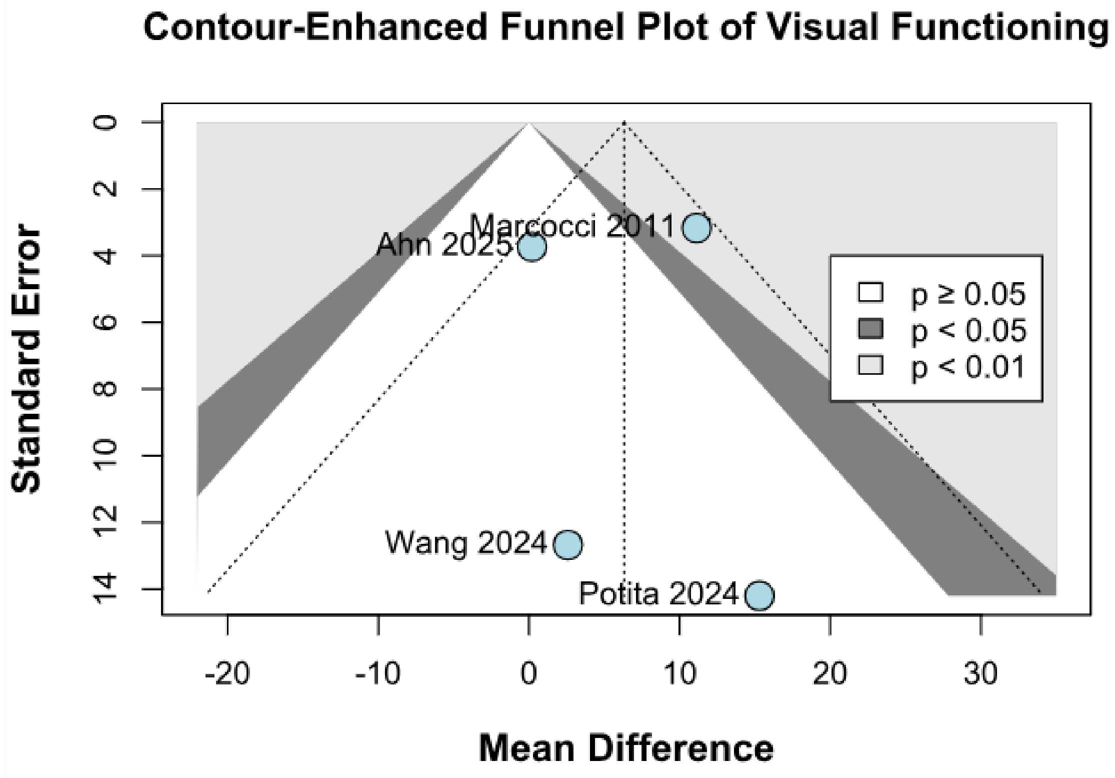

Figure S16. Contour-enhanced trim-and-fill funnel plot for visual functioning. The plot illustrates individual study weights against point estimates
